# Supplementary material for: Surface Microbiome Profiling of Dental Elevators Using Third‐Generation Sequencing: Implications for Infection Control in Dental Practice
Source: Microbiologyopen. 2025 Dec 8;14(6):e70178. doi: 10.1002/mbo3.70178 (PMC12685768; doi:10.1002/mbo3.70178)
Supplement: Supplementary file 1 — customer_backup. [file MBO3-14-e70178-s001.zip › customer_backup/customer_backup/taxa_summary/krona/samples/M16.Krona.html]

Javascript must be enabled to view this page.

members
magnitude
magnitudeUnassigned

M16.krona

61003

61003

12

12

12

12

12

12

1793

1793

1793

1793

1709

324

1332

53

38

38

46

46

710

86

86

1

1

1

85

14

14

71

71

3

2

2

2

2

1

1

1

1

621

512

512

49

44

5

463

9

11

181

74

11

30

35

5

8

99

7

5

4

4

1

1

2

1

1

1

1

73

73

73

55

3

15

12

12

9

9

3

3

17

17

1

1

16

16

2446

24

10

1

1

1

7

1

1

1

1

5

5

1

1

1

1

1

1

14

14

14

14

2422

206

2

2

2

204

9

9

1

1

179

126

26

27

15

15

2013

356

356

356

984

984

984

276

45

45

68

53

15

163

49

114

7

7

6

1

22

22

17

5

368

104

104

229

229

22

22

13

13

35

18

18

18

17

1

1

16

16

168

168

168

55

113

331

331

331

331

331

115

43

4

1

6

13

5

144

3972

3972

3972

3972

3972

458

3514

14046

14046

14046

13632

13632

13369

263

414

414

54

56

65

1

209

17

12

5

5

5

5

5

5

17400

7867

28

28

15

15

13

13

169

169

158

158

11

11

286

43

43

43

243

243

243

69

50

50

50

19

19

19

11

11

11

11

1112

976

38

1

37

20

8

1

11

14

14

14

14

116

76

7

33

4

4

44

28

1

15

1

1

65

63

2

12

1

11

2

2

67

66

1

13

13

6

6

108

108

107

107

38

38

3

1

2

3

3

2

2

69

5

55

8

1

230

80

150

136

136

136

355

278

21

7

14

12

12

20

15

5

175

1

39

135

50

50

17

17

17

43

20

20

23

23

17

17

17

5819

1674

101

29

72

437

437

437

423

14

36

36

663

235

59

227

142

3699

1

1

2295

2295

1113

1113

195

195

95

95

446

121

121

325

325

18

18

18

18

5344

75

19

1

1

18

18

56

32

32

24

1

1

22

1160

98

6

6

24

24

68

68

1062

1

1

3

3

79

79

14

14

1

1

8

8

934

27

907

22

22

154

93

93

93

61

61

61

1

1

1

1

3

3

3

3

6

6

6

6

3838

20

20

20

328

321

118

1

43

1

158

7

7

8

8

8

3482

2

2

15

12

2

1

2

2

2221

1

645

1575

3

3

5

5

738

738

491

491

1

1

3

3

1

1

107

107

107

73

34

4189

4189

1780

1780

594

154

59

11

520

9

152

281

2409

113

113

318

318

1967

1219

748

11

11

1596

1440

1440

1422

1422

1422

18

18

1

17

156

156

156

156

156

7247

4

4

4

4

4

7243

7243

4940

41

41

67

67

17

17

1758

1758

3057

3057

2303

2303

2303

1

1

1

1

1

1

11444

11444

694

694

694

227

433

23

11

10723

296

4

4

292

292

134

15

15

116

106

10

3

3

333

333

333

4

4

4

5586

5586

21

5563

2

244

244

90

6

2

28

18

31

69

1659

1581

1183

398

78

58

20

2208

66

66

113

113

72

72

262

262

30

30

1267

3

11

460

2

19

27

92

36

105

25

228

259

398

1

145

2

35

19

5

17

54

50

5

65

259

259

259

27

27

27

27
